# The effects of adaptation to urea on feeding rates and growth in *Drosophila* larvae

**DOI:** 10.1002/ece3.7770

**Published:** 2021-06-21

**Authors:** Kathreen Bitner, Grant A. Rutledge, James N. Kezos, Laurence D. Mueller

**Affiliations:** ^1^ Department of Ecology and Evolutionary Biology University of California, Irvine Irvine CA USA; ^2^ USDA HNRCA at Tufts University Boston MA USA; ^3^ Department of Development, Aging, and Regeneration Sanford Burnham Prebys Medical Discovery Institute La Jolla CA USA

**Keywords:** *Drosophila melanogaster*, energy, life‐history, trade‐offs

## Abstract

A collection of forty populations were used to study the phenotypic adaptation of *Drosophila melanogaster* larvae to urea‐laced food. A long‐term goal of this research is to map genes responsible for these phenotypes. This mapping requires large numbers of populations. Thus, we studied fifteen populations subjected to direct selection for urea tolerance and five controls. In addition, we studied another twenty populations which had not been exposed to urea but were subjected to stress or demographic selection. In this study, we describe the differentiation in these population for six phenotypes: (1) larval feeding rates, (2) larval viability in urea‐laced food, (3) larval development time in urea‐laced food, (4) adult starvation times, (5) adult desiccation times, and (6) larval growth rates. No significant differences were observed for desiccation resistance. The demographically/stress‐selected populations had longer times to starvation than urea‐selected populations. The urea‐adapted populations showed elevated survival and reduced development time in urea‐laced food relative to the control and nonadapted populations. The urea‐adapted populations also showed reduced larval feeding rates relative to controls. We show that there is a strong linear relationship between feeding rates and growth rates at the same larval ages feeding rates were measured. This suggests that feeding rates are correlated with food intake and growth. This relationship between larval feeding rates, food consumption, and efficiency has been postulated to involve important trade‐offs that govern larval evolution in stressful environments. Our results support the idea that energy allocation is a central organizing theme in adaptive evolution.

## Graphical abstract

Fifteen populations of fruit flies were raised with urea added to their larval food and 25 additional populations were raised without urea. These 40 populations show a wide range of larval feeding rates in part due to the evolution of slow feeding in response to urea. This work shows that there is a strong, positive correlation between larval feeding rates and larval growth rates.

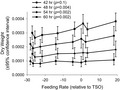

## 1 INTRODUCTION

An important focus of evolutionary research has been the elucidation of how phenotypes affect survival and reproduction (e.g., see Abrahamson & Weiss, 1997). Differential survival that results from phenotypic differentiation should then lead to changes in the genetic structure of populations (Lewontin, 1974). Making the connection between genes and phenotypes has been one of the more difficult challenges in evolutionary biology except for traits under simple genetic control. Advances in DNA sequencing technology have now made it possible to follow changes in the entire genome due to selection especially in laboratory‐selected populations (Schlötterer et al., 2014).

Recent research has shown inferring gene–phenotype relationships requires a large number of independent populations to achieve adequate statistical power (Baldwin‐Brown et al., 2014; Mueller et al., 2018). Most experimental evolution studies utilize just 6–10 independent populations. In this study, we have assembled a collection of forty independent populations to measure important phenotypes. We assessed the phenotypic differentiation that occurs in populations adapted to urea. Our results allowed us to determine the extent to which populations that have been selected for stress resistance or age‐at‐reproduction show correlated changes in the same phenotypes. Future work will collect genomic data from these forty populations to make gene–phenotype inferences.

The 40 study populations can be broken into three different categories. (a) Fifteen populations studied have been directly selected for tolerance to high levels of urea in their larval food. An additional five populations served as their direct controls. This group of twenty populations will be referred to as the urea selection populations. (b) Another 10 populations were derived from populations selected for late reproduction, five were subjected to desiccation stress and five served as controls under mild starvation stress. This collection of 10 populations will be referred to as the stress‐selected populations. (c) Finally, 10 populations were selected for late reproduction. These populations will be referred to as the demographically selected populations.

Selection for reproduction at later ages results in increased longevity as well as changes in a number of correlated traits (Partridge & Fowler, 1992; Service et al., 1985). Service et al. (1985) showed that populations selected for reproduction at later ages also showed increases in adult starvation resistance, desiccation resistance, and ethanol tolerance. Further evidence of these correlations came from populations directly selected for desiccation resistance and starvation resistance that also exhibited correlated increases in longevity (Rose et al., 1992). The stress‐selected populations may show high levels of starvation resistance and desiccation resistance due to their derivation from populations selected for postponed selection or their direct history of desiccation selection. The demographically selected populations would be expected to show stress resistance due to selection on these correlated traits. It is unclear whether larval stress selection will affect adult stress traits. We test that possibility here with the urea‐adapted populations.

Evolutionary studies using the model organism *Drosophila melanogaster* have often focused on the evolution of adult traits like behaviors (Mery & Kawecki, 2003), heat tolerance (Gilchrist & Huey, 1999), desiccation tolerance (Gibbs et al., 1997), starvation tolerance (Chippindale et al., 1996), and age‐specific survival and fecundity (Rose, 1984). A number of studies have examined the evolution of larval phenotypes like competitive ability (Mueller, 1988a), urea and ammonia tolerance (Borash et al., 2000), parasite tolerance (Fellowes et al., 1999), and low nutrition (Kolss et al., 2009). A common adaptation among the studies of larval adaptation is the evolution of larval feeding rates. Thus, crowded larval conditions result in the increase in larval feeding rates (Joshi & Mueller, 1988; although see also Nagarajan et al., 2016), while stresses like food laced with ammonia or urea (Borash et al., 2000) and larval parasites (Fellowes et al., 1999) result in decreased feeding rates.

Feeding rates have been shown to be correlated with larval competitive ability (Burnet et al., 1977). However, *Drosophila* larvae can evolve increased competitive ability without changing their feeding rate in certain environments (Nagarajan et al., 2016). There is also evidence that the rate at which food passes through the larval alimentary tract is proportional to the feeding rate (Burnet et al., 1977). Furthermore, larvae with very high feeding rates are less efficient than slower feeding larvae—that is they require more food to successfully pupate (Joshi & Mueller, 1996; Mueller, 1990). These findings suggested that energy trade‐offs may be driving the evolution of feeding rates (Mueller & Barter, 2015). An important foundation for theories of life‐history evolution is the concept of trade‐offs (Cody, 1966; Dudley & Schmitt, 1996; Gadgil & Bossert, 1970; Sinervo, 1990; Stearns, 1992). Cody (1966) originally developed the idea that an organism limited energy budget determined the currency of these trade‐offs. This idea was sufficiently powerful that entire fields, like optimal foraging, are based on the idea of maximizing energy intake (Charnov, 1976; MacArthur & Pianka, 1966). Adaptation of *Drosophila* larvae to stress offers rich experimental system to illustrate this idea.

Mueller and Barter (2015) suggested that in toxic food environments, a reduction in feeding rates would reduce the intake of toxins while increasing the efficiency of energy extraction from the food consumed. This would allow the larvae to route the extra energy they get from enhanced efficiency to detoxification. This theory hinges on the implied relationship between feeding rates and food consumption. This relationship has been called into question by Kaun et al. (2007) who found a weak positive but nonsignificant correlation between feeding rates and food consumption (see also Brown et al., 2019). In normal, nontoxic food, slowly feeding larvae should be below their optimum feeding rate, as measured by energy gained per unit time, and thus would be expected to grow more slowly than faster feeding larvae. We tested that prediction by measuring feeding rates and larval growth rates of all forty populations.

## 2 MATERIAL AND METHODS

### 2.1 Populations

**FIGURE 1:**
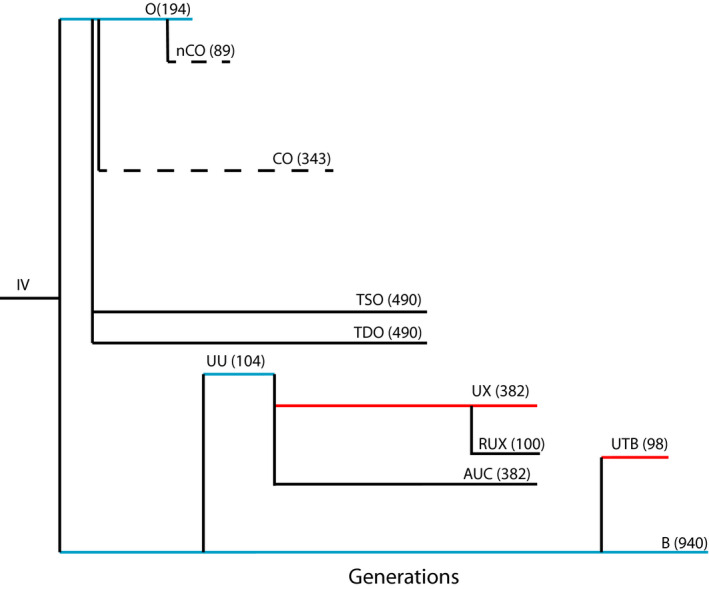
The phylogeny of laboratory‐selected populations used in this study. A selection regime is noted by a name like nCO or CO and each of these consists of five independent replicates. The numbers indicate the number of generations each selection regime has passed through at the time of the experiment. Solid black horizontal lines indicate populations on a three‐week generation cycle. Dashed horizontal lines indicate populations on four‐week generation cycles. Red lines indicate populations with urea added to the larval food. Blue lines indicate populations not studied in this experiment

Forty populations were used in this study from three different categories. The urea selection populations consisted of UX, RUX (reverse‐selected UX), and UTB selected for urea resistance and the control AUC (urea control). The stress‐selected populations consisted of the TDO (desiccation selected) populations which were originally selected for desiccation resistance in 1988 and the control TSO (desiccation controls) populations (Rose et al., 1992). The TSO populations experienced mild starvation selection. In 2005, the TSO and TDO populations were relaxed from selection and maintained on a 21‐day culture regime. At the time of these experiments, the TSO and TDO populations had been removed from selection for about 243 generations (Figure 1). The demographically selected populations consisted of the five CO and five nCO (new CO) populations. All populations are derived from an ancestral “IV” population collected from South Amherst, MA in 1975 by Philip Ives (Rose, 1984, Figure 1). After four and a half years of laboratory culture, the B_1–5_ populations (baseline, 14‐day generation cycle) and O_1‐5_ populations (70‐day generation cycle) were derived from the single IV population in 1980 (Rose, 1984; Rose et al., 2004). The CO_1–5_ populations (28‐day generation cycle) were derived from the O_1–5_ populations in 1989 (Rose et al., 1992). The nCO populations are a more recent creation of the CO selection regime.

All populations were maintained on a banana molasses food (Rose, 1984) at 25°C (24 hr light), at an uncontrolled humidity, and having a generation time of approximately 3–4 weeks. All selection regimes are fivefold replicated, uncrowded as larva (60–80 eggs per 8‐dram vial), with emergent adults kept at a low density of approximately 50–60 flies per 8‐dram vial, and transferred to a cage environment after 14 days of development from egg. Fresh food was provided in these cages about every other day for approximately 1 week. Effective population sizes in our experimental populations have an estimated range from 700–1,000 (Mueller et al., 2013) and are maintained at large breeding population sizes (≥1,000), with discrete generations.

The UX and the UTB populations were subjected to selection for increased larval tolerance to the presence of toxic levels of urea in the food for 382 and 98 generations respectively. Both selection regimes ultimately trace their ancestry back to the B populations (Figure 1). The level of urea was increased every few generations, when it was observed that a great proportion of larvae were surviving to adulthood. The final urea levels were 0.27 M. The urea‐tolerant (UX) and unselected controls (AUC) were derived in the fall of 1996. Both sets of populations were derived from the five UU populations, which had a 3‐week generation time, and were reared at low larval and adult densities (Figure 1). The UU populations were derived in 1990, from the Rose B populations (Chippindale et al., 1996, 1994; Rose, 1984). The RUX is a reverse‐selected line of the UX. The RUX populations have been in a control environment for 100 generations (Figure 1). The UTB line was created in October 2013 (Figure 1). The AUC, RUX, UX, and UTB lines are all 21‐day cycle flies. The TSO, TDO, nCO, and CO selection regimes did not include urea and were developed independently of the urea‐selected lines and their control.

For every assay, eggs were collected from the eight population types and passed through two generations of common, standard conditions—low larval density, 1,000 adult density, discrete generation times, 24‐hr light cycle, and regular banana molasses food (Bitner et al., 2020).

### 2.2 Feeding rates

Feeding rates were collected on all 40 populations. Eggs were collected from adults who had undergone a two‐generation standardization procedure. To measure the feeding rate, individual larvae around 48 hr old were gently moved onto a 3% agar coated with a 10% live yeast suspension. The larva was given 60 s to adjust to the new surroundings, and the sclerite retractions were recorded for 60 s. A total of twenty‐five larvae per population were individually measured this way. This procedure for measuring sclerite rates is similar to Sewell et al. (1975) and described in Joshi and Mueller (1988)

The feeding rates were measured in two blocks. The TSO populations were measured in each block and thus served as a reference. Feeding rates were standardized in each block as the difference from that block's TSO mean feeding rate. With this standardization, we let *y_ijk_* be the feeding rate difference for selection regime *i* (*i* = 1,…,7, since TSO is now used as a reference), population *j* (*j* = 1,…,35), and individual *k* (*k* = 1,…,25). The linear mixed effects model for *y_ijk_* is,

(1) $$y_{\mathrm{ijk}}=\mu +\alpha_{i}\delta_{i}+B_{j}+\varepsilon_{\mathrm{ijk}}$$

where *δ_i_* = 0, if *i* = 1, and 1 otherwise, *B_j_* and *ε_ijk_* are independent normally distributed random variables with zero mean and variances $\sigma_{B}^{2}$ and $\sigma_{\varepsilon }^{2}$. Random variation due to drift, founder effects, and handling of individual populations is reflected in *B_j_*, random variation between individual feeding rate measurements is measured by *ε_ijk_*. The model was analyzed with the linear mixed effects *R* function *lme* (R Core Team, 2017). Pairwise differences were evaluated after adjusting for multiple comparisons using Tukey's method implemented by the *R* function *lsmeans*.

The UX, RUX, and UTB populations all have a current or past history of being raised as larvae in food with urea. Previous research has demonstrated that feeding rates decline as populations adapt to urea (Borash et al., 2000). We were interested in testing the feeding rates of these three selection regimes as a group, and our initial statistical analysis showed no significant differences between UX, RUX, and UTB feeding rates. Thus, we pooled the feeding rate measurements for all 15 UX, RUX, and UTB populations into one urea population and repeated the analysis as described above comparing the pooled urea‐selected populations to the AUC control.

### 2.3 Starvation and desiccation

Individual female flies were collected from the eight selection regimes, a total of 30 flies per replicate per assay (150 flies per selection regime for one assay). Each fly was placed into a straw with pipette tips on both ends. The straws were wide enough for the flies to be able to move from one end to the other. The flies selected for the starvation assay were placed into straws with 3% agar while the flies selected for the desiccation assay were placed into straws with desiccant separated by cheese cloth. The cheese cloth functioned to prevent direct contact between the flies and desiccant. Flies subjected to desiccation were checked hourly, and flies undergoing starvation were checked every 4 hr.

Time to death by starvation and desiccation was collected on a total of eight selection regimes. We can let the desiccation (or starvation) time for selection regime *i* (*i* = 1,…,8), population *j* (*j* = 1,…,40), and individual *k* (*k* = 1,…,30) be *y_ijk_*. The linear mixed effects model for *y_ijk_* is,

$$y_{\mathrm{ijk}}=\mu +\alpha_{i}\delta_{i}+B_{j}+\varepsilon_{\mathrm{ijk}}$$

where *δ_i_* = 0, if *i* = 1, and 1 otherwise, *B_j_* and *ε_ijk_* are independent normally distributed random variables with zero mean and variances $\sigma_{B}^{2}$ and $\sigma_{\varepsilon }^{2}$.

### 2.4 Viability versus food type

The viability experiment started with 50 first instar larvae. However, due to the size of this experiment and technical difficulty of counting out exactly 50 larvae, the actual number of larvae put in each vial is more properly thought of as a random variable which could be both higher or lower than 50. Thus, in this analysis, we have analyzed the total number of larvae that survived to become adults under the assumption that the mean number of input larvae was the same in all treatments.

Larvae were raised under two experimental treatments, a control environment with regular food and an experimental environment of food with added urea (0.22 M urea). Ten vials were used per population per environment, for a total of 20 vials per population. Each vial was provided 50 first instar larvae. The total number of larvae which successfully eclosed as adults was recorded. Ultimately, we are interested in testing the effects of urea on survival for each population as well as differences between the different selection regimes. These experiments were also run in three blocks each separated by about one years’ time: (i) AUC, RUX, and UX, (ii) TSO, CO, nCO, and UTB, (iii) UTB, UX, CO, and nCO.

The analysis of the differences among the urea‐selected lines (RUX, UX, and UTB) and their control (AUC) was done with blocks (i) and (iii). For this analysis, viability was scaled to the mean viability of the UX populations in the urea food environment. The analysis of the stress populations and the demographically selected populations (TSO, CO, and nCO) was done with blocks (ii) and (iii). Viability for the stress and demographically selected populations was scaled to the mean viability of the UTB populations in urea.

Let *y_ijkm_* be the number of survivors in food type *i* (control (1), urea (2)), selection regime *j* (AUC (1), RUX (2), and UTB (3) in the urea analysis and TSO (1), CO (2), and nCO (3) in the demographic analysis), population *k* (*k* = 1,…,15), and replicate *m* (*m* = 1,…,10). Let ${\bar{y}}_{\text{urea}}$ be the mean viability in the urea control populations (UX for blocks (i) and (iii) and UTB for blocks (ii) and (iii)). We analyzed the differences, $\Delta_{\mathrm{ijkm}}=y_{\mathrm{ijkm}}‐{\bar{y}}_{\text{urea}}$. The effects of selection regime and food type were studied with the linear mixed effects model,

$$\Delta_{\mathrm{ijkm}}=\alpha +\delta_{i}\beta_{i}+\delta_{j}\gamma_{j}+\delta_{i}\delta_{j}\pi_{\mathrm{ij}}+b_{k}+c_{\mathrm{ijkm}}$$

where *δ_i_* = 0 if *i* = 1 and 1 otherwise, *b_k_* and *c_ijkm_* are the population and residual error terms respectively and assumed to have a mean of zero and different variances.

We also developed a statistical analysis of the relative level of adaptation to urea for each selection regime. Let *y*_urea_ *_jkm_* be the number of survivors in urea food, selection regime *j* (TSO, AUC, CO, nCO, RUX, UX, and UTB), population *k* (*k* = 1,…,35), and replicate *m* (*m* = 1,…,10). Then we analyzed the differences, $\Delta_{\mathrm{jkm}}={\bar{y}}_{\text{control}jk}‐y_{\text{urea}jkm}$ where ${\bar{y}}_{\text{control}jk}$ is the mean survival in control food in selection regime *j* and population *k*.

### 2.5 Developmental time versus food type

This assay looked at differences in the development time of the larvae. The measurements were made from the time first instar larvae were placed in vials. For each population, 10 vials were set up with 50 freshly hatched larvae each in a banana molasses environment. Another 10 vials were set up with urea food and 50 freshly hatched larvae. Eclosing adults were collected every 6 hr, separated by sex, and recorded.

The methods for the analysis of the development time experiment were the same as the viability analysis except there was one additional fixed effect, sex. The first analysis was on the difference between the urea development time and the control development time. Here, the mean control development time for each selection/sex/population/rep sample was calculated and then subtracted from the corresponding development time in urea.

To test for differences due to selection among urea populations, we analyzed blocks (i) and (iii) using UX‐urea as the standard. Thus, in block (i), we computed the mean UX‐urea development time, and this mean was subtracted from the other control and urea observations. The same was done for block (iii) using the UX‐urea mean from block (iii). To test the stress and demographically selected populations we analyzed blocks (ii) and (iii) using UTB as the standard. Thus, in block (ii) we computed the mean UTB‐urea development time, and this mean was subtracted from the other control and urea observations. The same was done for block (iii) using the UTB‐urea mean from block (iii).

### 2.6 Larval growth rate

Pairs of populations, matched by the replicate number, were assayed together in a randomized block design and the assay followed the same procedures as mentioned in Santos et al. (1997). With a two‐generation lead in for each population, 45 newly hatched first instar larvae were collected with a fine paint brush and placed onto non‐nutritive agar petri dishes with 3 ml of yeast paste (188 g of yeast in 500 ml of DI water) and placed randomly into a 25°C incubator with 24‐hr lighting. For each population, a separate petri dish of 45 larvae was created for every time sample in this experiment. There were 13 different “hour numbers” at which larvae were sampled: 24, 30, 36, 42, 48, 54, 60, 66, 72, 78, 84, 90, and 105 hr after the larvae were added to the petri dish. At the designated hour, 30 larvae were washed with DI water and then allowed to air dry. The wet weight, to the nearest 0.01 mg, of the 30 larvae was recorded before they were then placed into an 80 degree C drying oven, after which their dry weights were recorded.

The growth rates experiments were broken into three blocks due to the size of this experiment. The selection regimes assayed in each block were (a) AUC, CO, nCO, RUX, UTB, UX, (b) AUC, CO, RUX, UTB, UX, and (c) TSO, CO, TDO, and nCO. For each larval age and selection regime, there were between 5 and 13 samples of 30 larvae for a total of 751 samples of groups of 30 larvae.

Empirically, larval growth follows a logistic trajectory (Santos et al., 1997). We used a three‐parameter logistic function to model basic growth dynamics. Under this model, the size of individual larvae after *t*‐hours of growth is given by,

(2) $$f\left(\varphi ,t\right)=\frac{\varphi_{1}}{1+\exp\left[\left(\varphi_{2}‐t\right)/\varphi_{3}\right]}.$$

In model (2) the asymptotic maximum size is equal to *φ*_1_, and the time to reach half the maximum size is *φ*_2_. As t→∞, $\exp\left[\left(\varphi_{2}‐t\right)/\varphi_{3}\right]\to 0$, and the size approaches the asymptotic value *φ*_1_. The speed of this approach is governed by *φ*_3_. The smaller *φ*_3_ the faster the approach to the asymptote. With this model, we let *y_ijkt_* be the average size of a larva from selection regime *i* (*i* = 1 (AUC), 2 (CO), 3 (nCO), 4 (RUX), 5 (UTB), 6 (UX), 7 (TSO), and 8(TDO)), population *j* (*j* = 1,…,40), and block *k* at time *t*. Random variation arises due to both population effects (random genetic drift), block effects, and individual variation. Consequently, the size of larvae from selection regime *i* and population *j* at time *t* is *y_ijkt_* = *f*(**φ** *_ijk_*,*t*) + *ε_ijkt_*, and,

(3) $$\begin{array}{c} \varphi_{i1}=\alpha_{1}+\delta_{i}\gamma_{1i}\\ \varphi_{ijk2}=\alpha_{2}+\delta_{i}\gamma_{2i}+b_{j}+c_{k}\\ \varphi_{i3}=\alpha_{3}+\delta_{i}\gamma_{3i}, \end{array}$$

where *δ_i_* = 0, if *i* = 1 and 1 otherwise. The within‐population variation, *ε*, is assumed to be normally distributed with a zero mean. This variation increases as the larvae get larger so we assumed that Var(*ε*) = $\sigma^{2}{\left|t\right|}^{2\Delta }$ where ∆ is estimated from the data. Population variation, *b*, and block variation, *c*, is assumed to only affect parameter *φ*_2_. We tested models with population variation in the other parameters, and the model with variation in *φ*_2_ was chosen due to having the lowest Akaike and Bayesian information criterion (Pinheiro & Bates, 2000, chapter 8). The population variation is assumed independent of the within‐population variation and has a normal distribution with zero mean and variance, $\sigma_{b}^{2}$. Parameters of Equation (3) were estimated by the restricted maximum likelihood techniques implemented by the *nlme* function in R (R Core Team, 2017).

When displaying the predicted larval size from Equations (2 and 3), we also calculated 95% confidence intervals. With eight different selection regimes, we have 24 maximum likelihood parameters estimates and their covariance matrix estimates, $\hat{\mu }=\left({\hat{\alpha }}_{1},{\hat{\alpha }}_{2},{\hat{\alpha }}_{3},{\hat{\gamma }}_{12},{\hat{\gamma }}_{22},{\hat{\gamma }}_{32},\ldots ,{\hat{\gamma }}_{38}\right)$ and $\hat{\Sigma }$. These were assumed to have a *t*‐distribution. From these distributions, we drew samples of the parameter vectors, ${\tilde{\mu }}_{k}$, (*k* = 1,…,*m*). For each sampled parameter vector, we made size predictions for each selection regime for ages, 42, 48, 54, and 60 hr. At a specific age, let the *k*th (out of *m*) prediction for selection regime *i* be ${\tilde{y}}_{\mathrm{ki}}$. From these *m* predictions, we generated order statistics, $\Delta^{s}\left({\tilde{y}}_{\mathrm{ki}}\right)$, where $\Delta^{1}\left({\tilde{y}}_{\mathrm{ki}}\right)$ is the smallest predicted value at *t* and $\Delta^{m}\left({\tilde{y}}_{\mathrm{ki}}\right)$ is the largest. From the order statistics, we then used $\Delta^{l}\left({\tilde{y}}_{\mathrm{ki}}\right)$ as the lower confidence limit and $\Delta^{u}\left({\tilde{y}}_{\mathrm{ki}}\right)$ as the upper confidence limit. In our simulations, we set *m* = 5,000. Therefore, a 95% confidence interval corresponds to $\Delta^{l}\left({\tilde{y}}_{\mathrm{ki}}\right)=\Delta^{125}\left({\tilde{y}}_{\mathrm{ki}}\right)$ and $\Delta^{u}\left({\tilde{y}}_{\mathrm{ki}}\right)=\Delta^{4876}\left({\tilde{y}}_{\mathrm{ki}}\right)$.

One hypothesis of interest was whether there was a relationship between the larval feeding rates and the growth of larvae. To test this, we fit a line to the larval size versus feeding rate observations at the four larval ages around 48 hr—the age our feeding rates were measured. A significant positive slope for these lines was taken as evidence consistent with our hypothesis.

An alternative method of analysis would have been to use the actual observed size of larvae for each selection regime and larval age. However, the advantage of using the predictions is that a much larger sample is utilized to estimate the three‐parameter model (Equation 2). The logistic model is an excellent empirical model for this biological process (see Figure 12). Alternatively, using individual estimates of larval size for each selection regime would rest on only five replicates for some selection regimes.

### 2.7 Adult size

The adult size was collected from the same larvae collected for the larval growth rate assay (Section 2.6). Following Santos et al. (1997), at hour 105, 30 pupae were collected and placed into non‐nutritive agar vials to allow for their development. When they had eclosed, FlyNap was used to anesthetize the flies to record their wet weights. Afterward, they were placed into a drying oven at 80 degrees Celsius for 48 hr. The dry weight of the adult flies was then recorded after the 48 hr had passed. The statistics for analyzing the adult size of flies are the same as the development time differences.

## 3 RESULTS

### 3.1 Larval feeding rates

**FIGURE 2:**
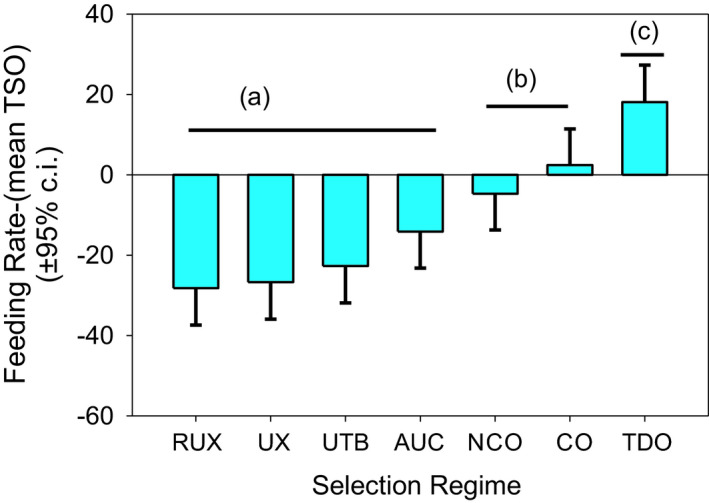
Larval feeding rates relative to the TSO populations measured as sclerite contractions per minute. The horizontal bars mark groups that are not significantly differentiated form each other. Between the separate groups are significant differences, with TDO feeding the fastest and RUX feeding the slowest

The RUX, UX, and UTB populations fed at similar rates and are not significantly different from each other (Table 1, Figure 2). A significantly slower feeding rate was observed in the urea‐adapted lines—UX, UTB, and RUX—compared to the stress line TDO (*p* < .0001) and the demographic line CO (*p* ≤ .0013). RUX and UX fed significantly slower than nCO (*p* ≤ .024) but UTB did not.

**TABLE 1:** Mean larval feeding rates (sclerite retractions per minute), sample size (*n*), and 95% confidence intervals. The means are based on samples from all blocks

Selection regime	*n*	Mean	95% confidence interval
AUC	125	90.6	87.9, 93.4
TSO	250	114.0	112.0, 116.0
CO	250	116.0	114.0, 118.0
TDO	125	141.0	139.0, 144.0
nCO	250	109.0	106.0, 112.0
RUX	125	76.6	74.0, 79.2
UTB	125	82.1	79.9, 84.3
UX	125	78.0	74.7, 81.3

**FIGURE 3:**
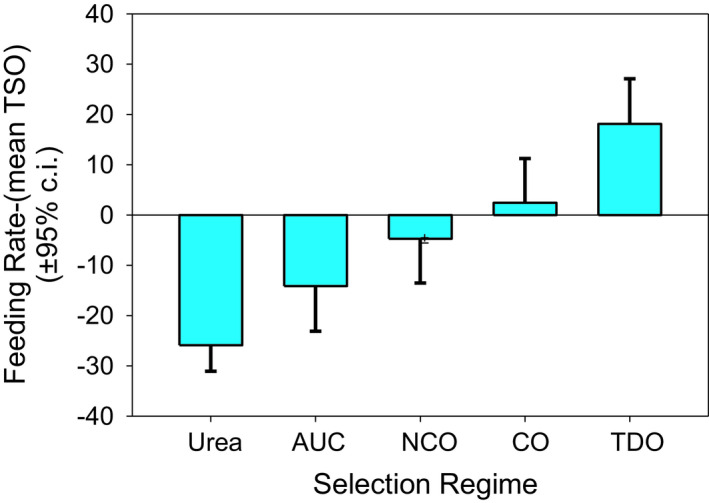
A one‐sided test comparing the urea‐pooled populations (UX, UTB, and RUX) against the AUC control, nCO, CO, TSO, and TDO. The mean feeding rate difference for the fifteen urea populations was −25.88. This is significantly less than the AUC feeding rate difference (−14.13) with *p* = .014. The urea populations also fed significantly slower than the nCO, CO, TSO, and TDO

The pooled UX, RUX, and UTB showed slower feeding rates than the AUC controls (*p* = .014; Figure 3).

### 3.2 Starvation and desiccation resistance

**FIGURE 4:**
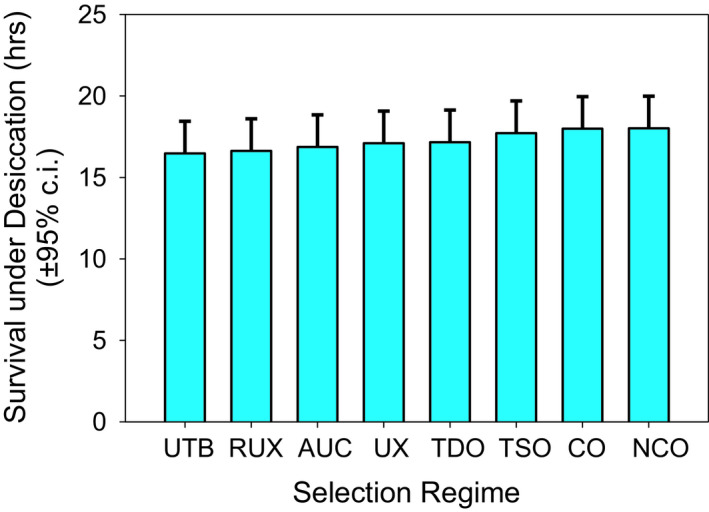
Mean desiccation times for 40 populations in eight different selection regimes. No significant difference was observed between any of the selection regimes in their resistance to desiccation

**FIGURE 5:**
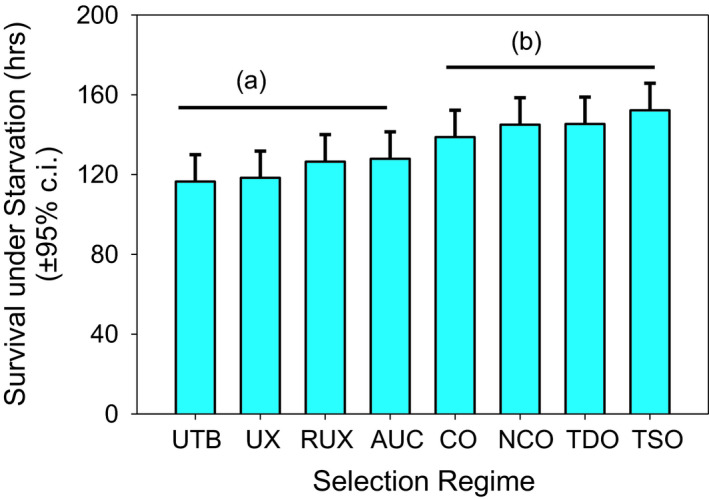
Mean starvation times for 40 populations in eight different selection regimes. No difference between the selection regimes under the horizontal bars, but significant difference was seen in some comparisons. UTB had a significant difference between TSO, TDO, NCO, and CO. UX had a significant difference between TSO, TDO, and NCO. RUX and AUC had a significant difference with TSO

Two of the selection regimes, TSO and TDO, had undergone selection in their evolutionary history for resistance to starvation (TSO) and starvation and desiccation (TDO) but were now relaxed from selection and maintained on a 21‐day culture regime. There was no significant difference in desiccation resistance between any of the eight selection regimes (Table 2, Figure 4). Starvation resistance showed no difference between the selection regimes under the horizontal bars (RUX, UX, UTB, and AUC vs. nCO, CO, TDO, and TSO), but significant differences were seen in some selection regime comparisons (Table 3, Figure 5). Starvation times were significantly shorter for the UTB populations compared to TSO (36 hr, *p* = .0002), TDO (29 hr, *p* = .0026), nCO (28 hr, *p* = .0029), and CO (22 hr, *p* = .039). Starvation times were significantly shorter for UX populations compared to TSO (34 hr, *p* = .0003), TDO (27.0 hr, *p* = .0054), and nCO (26.6 hr, *p* = .0061). TSO populations also showed significantly greater starvation times than the RUX (26 hr, *p* = .0090) and AUC populations (24 hr, *p* = .0016).

**TABLE 2:** Mean desiccation times (time to death from desiccation in hours), sample size (*n*), and 95% confidence intervals

Selection regime	*n*	Mean	95% confidence interval
AUC	146	16.9	16.4, 17.4
TSO	149	17.7	17.1, 18.4
CO	150	18.0	17.4, 18.5
TDO	150	17.2	16.6, 17.7
nCO	150	18.0	17.5, 18.5
RUX	150	16.6	16.1, 17.2
UTB	150	16.5	16.0, 17.0
UX	150	17.1	16.5, 17.7

**TABLE 3:** Mean starvation times (time to death from starvation in hours), sample size (*n*), and 95% confidence intervals

Selection regime	*n*	Mean	95% confidence interval
AUC	141	128	123, 133
TSO	144	152	147, 157
CO	145	139	134, 144
TDO	147	145	141, 150
nCO	148	145	140, 150
CO	146	126	121, 132
UTB	147	117	112, 121
UX	150	118	113, 123

### 3.3 Viability in urea

**FIGURE 6:**
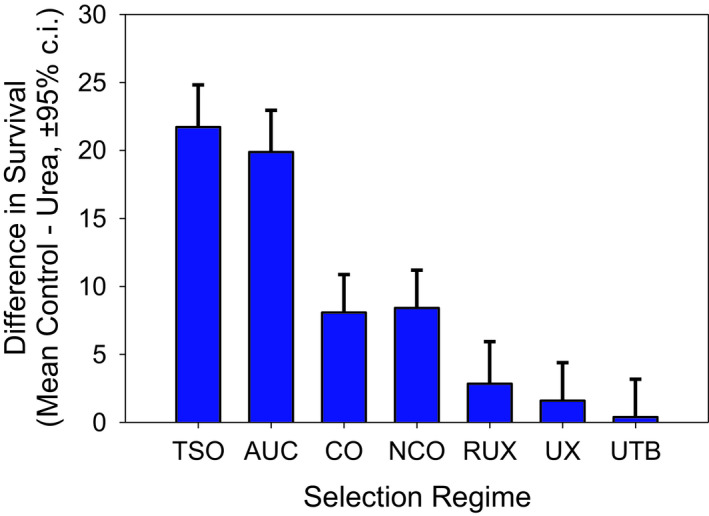
Difference in survival subtracting number of survivors in urea food from the number of survivors in control food. The TSO, AUC, CO, and nCO differences are significantly different from zero. The bars are 95% confidence intervals. The difference of AUC is significantly greater than RUX, UX, and UTB in each case with a *p* < .0001

We compared the viability (number of larvae surviving to adult stage) of each population in the control environment to the urea environment by computing the difference in the two (Table 4, Figure 6). If this difference is positive and significantly different from zero, it indicates sensitivity to the toxic effects of urea. The TSO, AUC, CO, and nCO populations show significant sensitivity to urea (Figure 6). The viability difference was not significantly different from zero in the urea‐adapted populations, RUX, UX, and UTB populations.

**TABLE 4:** Mean viability (number of eclosed adults from a sample of 50 first instar larvae), sample size (*n*), and 95% confidence intervals in the control and urea environment

Selection regime	Food type	*n*	Mean	95% confidence interval
AUC	Control	50	47.9	47.1, 48.8
AUC	Urea	50	28.0	26.6, 29.4
TSO	Control	50	44.2	42.5, 46.0
TSO	Urea	50	22.5	20.7, 24.3
CO	Control	100	46.6	45.0, 48.1
CO	Urea	100	38.5	37.1, 39.9
nCO	Control	100	45.8	44.5, 47.1
nCO	Urea	100	37.4	36.0, 38.7
RUX	Control	50	43.4	42.2, 44.7
RUX	Urea	50	40.6	39.3, 41.9
UTB	Control	100	46.8	45.7, 47.8
UTB	Urea	100	46.4	45.4, 47.4
UX	Control	96	44.6	43.6, 45.7
UX	Urea	100	43.0	42.1, 44.0

Note

The means are based on samples from all blocks. Note the sampling units are vials of 50 first instar larvae.

**FIGURE 7:**
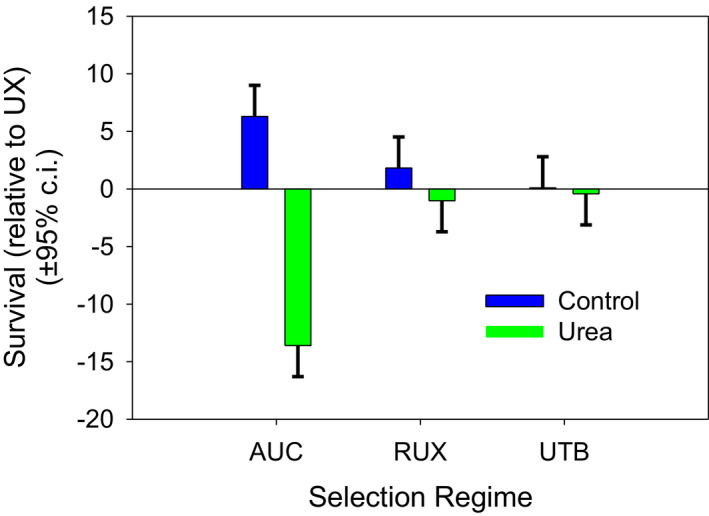
Survival of the urea lines (RUX, UTB, and UX) and control (AUC) in urea and control food. Survival is shown relative to the UX‐urea mean survival in each block. This makes the mean UX survival in urea 0 and in the control environment, 1.5. In the control environment, AUC survival was not significantly different from UX and RUX, but was significantly greater than UTB. In the urea environment, AUC survival is significantly less than UX, RUX, and UTB

**FIGURE 8:**
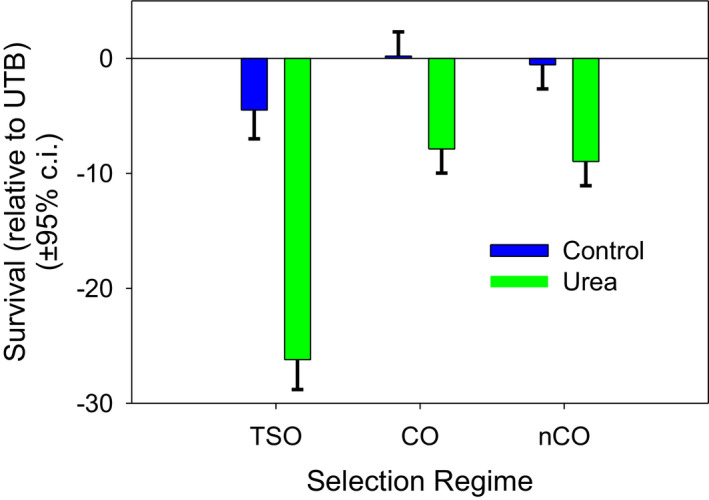
Survival of the demographic lines (TSO, CO, and nCO) in urea and control food. Survival is shown relative to the UTB‐urea mean survival in each block. This makes the mean UTB survival in urea 0 and in the control environment, 0.39. In both environments, the TSO selection regime has lower viability than the CO and nCO regimes. There are no significant differences between the CO and nCO regimes in either environment

The viability of the AUC populations in the control environment was not significantly greater than UX and RUX populations, but was significantly greater than UTB (*p* = .022, Figure 7). In the urea environment, AUC viability was significantly less than UX (*p* < .001), RUX (*p* < .001), and UTB (*p* < .001, Figure 7). In the control environment, the TSO selection regime had lower viability than the CO regime (*p* = .04) but not the nCO regime. In the urea environment, the TSO selection regime had lower viability than the CO regime (*p <* .0001) and the nCO regime (*p* < .0001). There were no significant differences between the CO and nCO regimes in either environment (Figure 8).

### 3.4 Development time in urea

**FIGURE 9:**
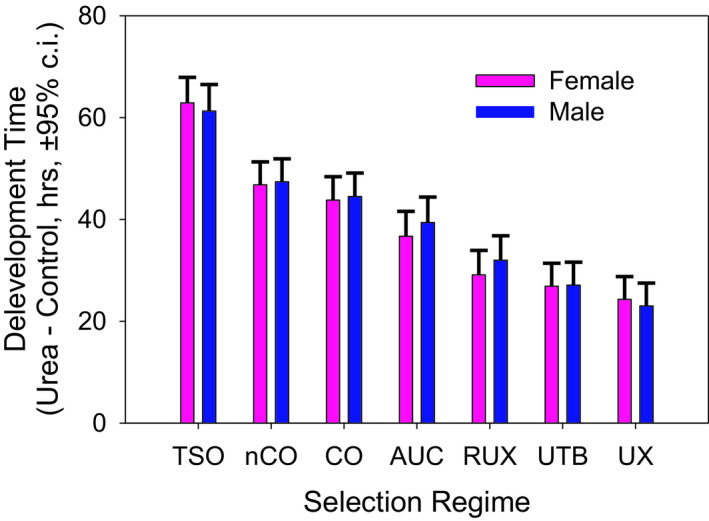
Development times of each population in urea food relative to the development time in control food for seven selection regimes—TSO, nCO, CO, AUC, RUX, UTB, and UX

The developmental time (Table 5) is calculated as the time it took for the first instar larvae to pupate and eclose. The first analysis examined the difference between the development time in urea food and the control development time (Figure 9). Here, the mean development time in the control environment for each selection/sex/population/replicate sample was calculated and then subtracted from the corresponding development time in urea. Thus, a positive value for this difference indicates that the larva takes longer to develop in urea. The populations that have been selected for increased larval tolerance to urea, RUX, UX, and UTB, show the smallest development time difference consistent with their adaptation to urea (Figure 9). However, all populations show a development time difference that is positive and significantly different than zero (see confidence intervals in Figure 9). All populations, even populations adapted to urea, show delayed development in urea‐laced food.

**TABLE 5:** Mean development time (hr), sample size (*n*), and 95% confidence intervals in the control and urea environment. The means are based on samples from all blocks

Selection regime	Food type	*n*	Mean	95% confidence interval
Females				
AUC	Control	1,400	241.9	241.3, 242.5
AUC	Urea	802	278.4	276.7, 280
TSO	Control	1,267	257	256.2, 257.8
TSO	Urea	666	319.5	316.8, 322.3
CO	Control	2,915	225.4	224.9, 225.8
CO	Urea	2,206	269.8	268.7, 270.9
nCO	Control	2,795	222.9	222.5, 223.3
nCO	Urea	2,047	269.8	268.6, 270.9
RUX	Control	1,207	250.8	250.1, 251.6
RUX	Urea	1,078	280.7	279.4, 282
UTB	Control	2,728	244.7	243.8, 245.5
UTB	Urea	2,607	272	270.6, 273.4
UX	Control	2,499	238.6	237.9, 239.2
UX	Urea	2,252	263	262.1, 263.9
Males				
AUC	Control	1,096	245	244.4, 245.7
AUC	Urea	698	283.8	281.9, 285.7
TSO	Control	1,043	262	261, 263
TSO	Urea	555	323.1	319.7, 326.5
CO	Control	2,259	229.8	229.2, 230.3
CO	Urea	1,942	274.2	272.9, 275.4
nCO	Control	2,321	228	227.4, 228.5
nCO	Urea	1,992	275	273.7, 276.2
RUX	Control	1,065	254.2	253.4, 255
RUX	Urea	1,049	286.2	284.8, 287.7
UTB	Control	2,518	247.9	247, 248.8
UTB	Urea	2,371	275.8	274.4, 277.3
UX	Control	2,256	241.6	240.9, 242.3
UX	Urea	2,350	265.3	264.3, 266.2

**FIGURE 10:**
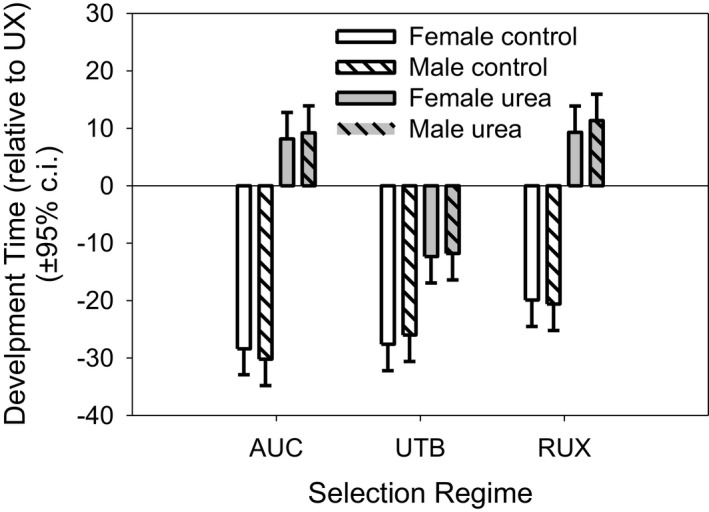
Relative development times for the urea selection regimes (RUX, UTB, and UX) and their control (AUC). Development time, in hours, is shown relative to the UX‐urea mean development time in each block. Thus, fast developing populations have large negative times and slow developing populations have large positive values

We assessed differences in development time among urea‐selected populations separately from stress and demographically selected populations. The actual mean development times in urea for the relevant UX populations were: UX‐female (block‐i) 270.7 hr, UX‐male (block‐i) 274.7 hr, UX‐female (block‐iii) 255.5 hr, UX‐male (block‐iii) 257.4 hr. For the control environment: UX‐female (block‐i) 247.8 hr, UX‐male (block‐i) 251.7 hr, UX‐female (block‐iii) 229.6 hr, UX‐male (block‐iii) 232.9 hr. Thus, in the urea environment the UX development time difference is 0, since $\sum_{i=1}^{n}(x_{i}‐\bar{x})=\sum_{i=1}^{n}x_{i}‐n\bar{x}=n\bar{x}‐n\bar{x}=0$. In the control environment, the UX development time difference is −24.4 (females) and −23.75 (males). We found that in the urea environment, AUC females (20.5 hr, *p* < .0001) and males (21.0 hr, *p* < .0001) had significantly longer development times than their respective UTB sexes, but there were no significant differences between the UX or RUX selection regimes (Figure 10). In the control environment, the development time of the AUC females was not significantly greater than UTB, RUX, or UX, while AUC male development times were significantly less than RUX (9.6 hr, *p* = .045), but not different than UX or UTB.

**FIGURE 11:**
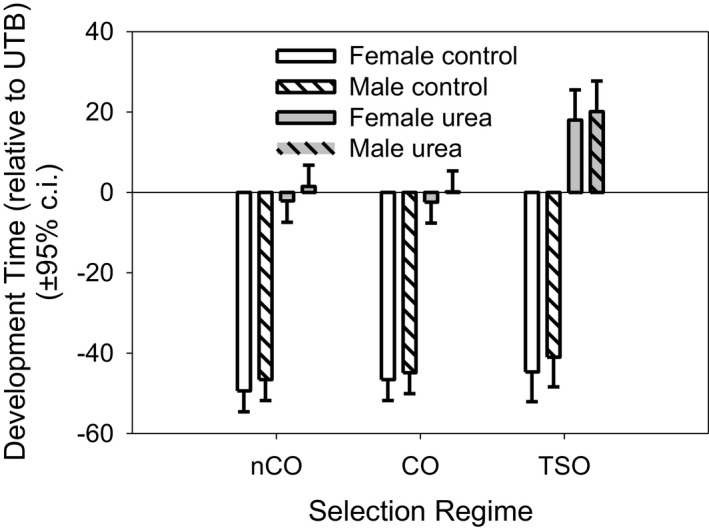
Relative development time for the demographic regimes (nCO, CO, and TSO). Development times are shown relative to the UTB‐urea mean development time in each block for the demographic lines. Thus, fast developing populations have large negative times and slow developing populations have large positive values

Next, we analyzed the effects of urea on the stress and demographically selected populations. The actual mean development times for the relevant UTB populations, which were used as the standard, were as follows: Females: UTB‐urea (block‐ii) 301.8 hr, UTB‐urea (block‐iii) 243.0 hr; Males: UTB‐urea (block‐ii) 302.8 hr, UTB‐urea (block‐iii) 245.8 hr; for the control environment: Females: UTB‐control (block‐ii) 262.5 hr, UTB‐control (block‐iii) 227.9 hr; Males: UTB‐control (block‐ii) 266.0 hr, UTB‐control (block‐iii) 231.4 hr. In the control environment, there are no significant differences between males and females from the CO, nCO, and TSO populations (Figure 11). In the urea environment, the TSO females developed significantly slower than the CO (20.4 hr, *p* = .0012) and nCO (20.1 hr, *p* = .0014) flies. Likewise, the TSO males developed more slowly than the CO (19.9 hr, *p* = .0016) and nCO (18.6 hr, *p* = .0029) flies. There were no significant differences between the nCO and CO flies.

### 3.5 Larval growth rates

**FIGURE 12:**
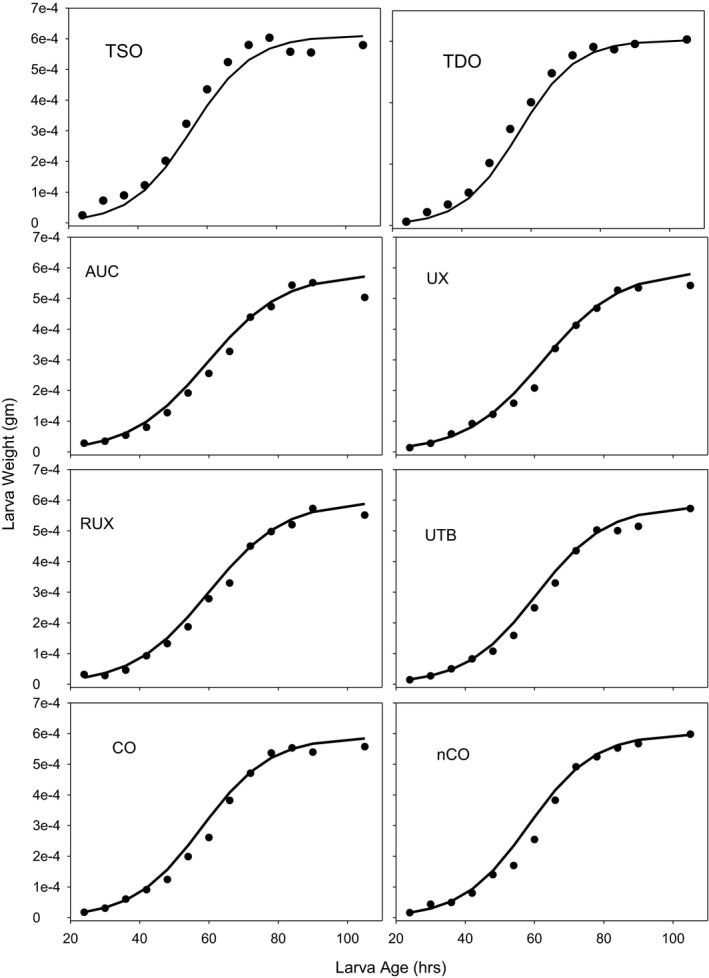
The mean dry weight of larvae at various ages for all eight populations—TSO, TDO, AUC, UX, RUX, UTB, CO, and nCO. The points are the average weights over the five replicate populations. The lines are the predicted weights from Equation (2)

The larval growth rate assay provided dry weight of larvae for all eight selection regimes—TSO, TDO, AUC, UX, RUX, UTB, CO, and nCO at hours 24, 30, 36, 42, 48, 54, 60, 66, 72, 78, 84, 90, and 105. The pupae that were collected at the 105‐hr mark were weighed and dried. A separate collection of pupae was collected for the adult weights. The mean dry weights of each selection regime and the fitted growth equation (Equation 2) are shown in Figure 12. The asymptote seen in all populations between hours 90 and 105 represents the transition of larvae from an actively feeding stage to a wandering stage during which they search for a pupation site. The cessation of feeding thus leads to the plateau in larval size.

**FIGURE 13:**
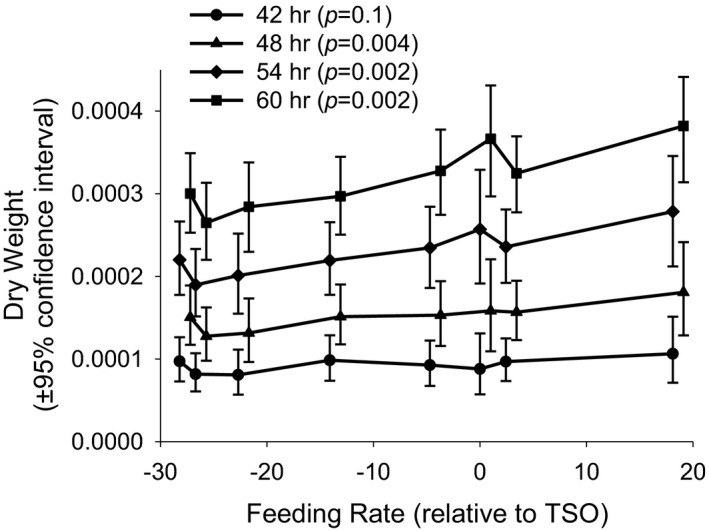
The predicted individual larval dry weight, with 95% confidence intervals, as a function of feeding rate. The *p*‐values are for tests of the hypothesis that the slope of the line is zero. The order of the eight selection regimes along the *x*‐axis is from slowest feeding to fastest: RUX, UX, UTB, AUC, nCO, TSO, CO, and TDO. The *x*‐axis coordinates are offset to improve readability

The predicted sizes of larvae from Equation (2) were used to estimate the linear relationship between larval size and feeding rate for different aged larvae (Figure 13). The order of the eight selection regimes in Figure 13 from slowest feeding to fastest was RUX, UX, UTB, AUC, nCO, TSO, CO, and TDO. A significant correlation was seen at hours 48 (*p* = .004), 54 (*p* = .002), and 60 (*p* = .002) but not 42 hr. The 46–60‐hr results support the conclusion that a slower feeding rate results in a slower growth rate.

## 4 DISCUSSION

The three selection regimes that have a history of exposure to urea in the larval environment (RUX, UX, and UTB) all show adaption to that environment in the form of increased viability in urea‐laced food. They also show reduced feeding rates as has been previously documented (Borash et al., 2000). One difference between RUX, UX, and UTB populations is their development times in urea‐laced food. Populations from the UX and UTB selection regimes show reduced development times relative to the AUC control, although only the UTB difference was significant, while populations from the RUX selection regime do not. The RUX populations were kept in control environments for 100 generations prior to these experiments but have continued to retain some of their adaptations to urea. One reasonable explanation for lack of convergence is the fixation of alleles at important loci which affect both survival in urea and larval feeding rates. Upon return to the control environment, the fixed alleles do not revert to the ancestral frequency.

Occasionally selection on one life‐stage has resulted in phenotypic differentiation in another life stage. Chippindale et al. (1994) show that adult selection on age‐at‐reproduction may affect egg‐to‐adult development time. Mery and Kawecki (2003) have shown that selection for adult learning has resulted in differentiation in larval survival at different food levels. The adaptation of the RUX, UX, and UTB selected lines to urea stress does not appear to confer adaptation to the adult stresses of starvation and desiccation. Next, we address if selection on adult traits has conferred any adaptation of larvae to resist urea. Certainly, the TSO, nCO, and CO populations do not have the level of urea resistance exhibited by the urea‐adapted populations, RUX, UX, and UTB. However, there are differences between the demographic populations, nCO and CO, and the stress‐selected population TSO. The demographic populations both survive better and develop more rapidly than the TSO populations in urea. In fact, the difference in the survival of nCO and CO populations in urea food and normal food is less than the AUC populations (Figure 6). As noted in the introduction, demographic selection has resulted in increased stress resistance in previous experiments (Service et al., 1985). The nCO and CO populations are on a four‐week generation cycle, while all other populations are on a three‐week cycle and show some partial resistance to urea.

None of the populations show any differences in their ability to resist desiccation. Thus, the past desiccation resistance of the TDO populations has been lost during their 243 generations of reverse selection. The stress‐selected and demographic selection populations all show similar starvation resistance and are superior to the urea‐selected populations and their control. Elevated starvation resistance for the demographic populations is not surprising given previous documentation of increased starvation resistance as a correlated trait to selection for later life reproduction. However, the increase in starvation resistance of the stress‐selected lines is more difficult to explain given their 243 generations of reverse selection. One possibility is that the genetically based increase in starvation resistance that was present in the original founding O populations or the by‐product of desiccation and weak starvation selection has resulted in allele fixation for favorable alleles which cannot be easily removed even after 243 generations in a control environment.

To understand the selection on feeding rates, we focus our discussion on three environments, (a) crowded environments with extreme competition for food, (b) environments with toxins in the food like ammonia or urea, and (c) environments with neither toxins nor extreme competition. In crowded environments, larvae may increase their competitive ability by increasing their feeding rates (Guo et al., 1991; Mueller, 1988a, 1988b; Mueller et al., 2005). In addition, increased feeding rates appear to be correlated with increased foraging path lengths which in crowded environments may assist larvae in finding less crowded food resources (Mueller et al., 2005; Sokolowski et al., 1997). We note that there are complications to this simple view of the effects of crowding on feeding rates, but this does represent one possible outcome of evolution (Borash et al., 1998; Nagarajan et al., 2016). None of the populations used in this study have been subject to crowding so hence this particular selective pressure is not believed to have impacted any of the study populations.

Environments with toxins, like urea or ammonia, result in declining feeding rates (Borash et al., 2000; Mueller et al., 2005). Reduced feeding rates will reduce the intake of toxic compounds which the larva must then detoxify. Reduced feeding rates also increase the efficiency of energy extraction (Mueller, 1990), thus facilitating the energy‐consuming process of detoxification (Mueller & Barter, 2015). In both environments, crowding (Mueller & Joshi, 2000, figure 6.30) and toxins (Borash et al., 2000), we see a rapid change in feeding rates, at least in laboratory conditions, suggesting strong selection for these phenotypic changes in feeding rates. Of course, the RUX, UX, and UTB populations have all been subject to the selective pressures of urea detoxification and as seen in this study and elsewhere have all evolved slower feeding rates.

The third environment, with no or little competition and no toxins, is the one experienced by the AUC, nCO, CO, TSO, and TDO populations. Given the abundance of food and the very weak pressure on fast development, we would expect selection to act on only the most extreme very slow or very fast feeding rates. Under these conditions, it would not be unexpected to find a wide range of feeding rates and a wide range of growth rates, during the larval stages.

Our results have shown that larvae between 48 and 60 hr show a positive relationship between feeding rates and larval dry weight although the result at 42 hr is not significant. Thus, at 60 hr an increase in feeding rate of 10 retractions per minute will increase dry weight from 5.9% (at the highest feeding rate) to 8.1% (at the lowest feeding rate). The feeding rates are measured using 48‐hr‐old larvae. This conclusion is different from Kaun et al. (2007) who found no statistically significant correlation between feeding rates and food ingestion. There are three possible explanations for the difference between Kaun et al.'s results and our own listed as follows: (i) no physiological relationship exists between feeding rates and ingestion or growth—the observed statistical relationship is artifactual—(ii) a positive relationship exists but the Kaun et al. experiments lacked the power to detect that relationship, or (iii) no relationship exists between feeding rates and ingestion but there is a positive correlation between feeding rates and larval growth rates. The third explanation is very difficult to explain especially since it stands to reason that faster feeding larvae would require more energy for this activity. So, we exclude explanation (iii) as an unreasonable alternative. We believe the second explanation is correct which we explain next.

Kaun et al. studied a total of 73 individuals whose feeding rate varied from 48 to 75 retractions per minute. Their data showed a positive relationship but with a *p*‐value of only .11. In the present study, feeding rates of 1,375 individuals were measured with a range of 10 to 169. Selection regimes used in our study had mean feeding rates that varied from 79 to 141. Thus, not only was our sample size larger by a factor of almost 20, but also the range of feeding rates was much larger. Thus, we believe our analysis has much greater power to detect a correlation between feeding rates and growth due to both the increased sample size and greater range of feeding rates. To test this idea, we generated computer samples to recreate Figure 13. We restricted our database to feeding rates between 114 and 141. This included all selection regimes except RUX. We sampled only 73 individuals from all populations except TSO to estimate mean feeding rates on the *x*‐axis of Figure 13. We used all of the growth rate data to create the same *y*‐axis values as used in Figure 13. The TSO population was always added to these computer‐generated data since TSO was the standard. For each of the four time intervals shown in Figure 13 (42, 48, 54, and 60 hr), we created 100 independent samples to test for significant slopes. The fraction of 100 tests that were significant was 8%, 0%, 0%, and 0% at hours 42, 48, 54, and 60 respectively. These results support our contention that small sample sizes and a limited range of feeding rates would obscure the trend we observed.

This study revealed extensive variation among the forty populations for larval feeding rates, viability and development time in urea, and starvation resistance. However, there was little variation for desiccation resistance. Computer simulations have shown that gene–phenotype relationships are more likely to be uncovered for phenotypes with large levels of genetic variation relative to environmental variation (Mueller et al., 2018). Thus, except for desiccation resistance, we believe genomic studies of these populations should yield insights into gene–phenotype connections.

## 5 CONCLUSIONS

Populations subject to demographic selection show elevated resistance to starvation relative to populations selected for urea resistance. These demographic populations may also show partial resistance to urea in their larval food. Selection for urea resistance does not enhance adult stress resistance against starvation or desiccation. The results of this study provide convincing evidence of the relationship between larval feeding rates and larval growth rates. This relationship suggests that energy allocation to activities like growth and detoxification of urea are central to the evolution of feeding rates as suggested by Mueller and Barter (2015). In addition, they point to a way of understanding the central role feeding rates have played in the evolution of *Drosophila* larvae to a wide array of challenging environments.

## CONFLICT OF INTEREST

None declared.

## AUTHOR CONTRIBUTIONS

**Kathreen Bitner:** Investigation (lead); Project administration (lead); Writing‐original draft (lead). **Grant A. Rutledge:** Investigation (supporting); Writing‐review & editing (supporting). **James N**. **Kezos:** Investigation (supporting); Writing‐review & editing (supporting). **Laurence D. Mueller:** Formal analysis (lead); Supervision (lead); Writing‐review & editing (lead).

## Data Availability

The data can be accessed at: https://doi.org/10.7280/D1VT3G.

## References

[ece37770-bib-0001] Abrahamson, W. G. , & Weis, A. E. (1997). Evolutionary ecology across three trophic levels. Princeton University Press.

[ece37770-bib-0002] Baldwin‐Brown, J. G. , Long, A. D. , & Thornton, K. R. (2014). The power to detect quantitative trait loci using resequenced, experimentally evolved populations of diploid, sexual organisms. Molecular Biology and Evolution, 31, 1040–1055. 10.1093/molbev/msu048 24441104 PMC3969567

[ece37770-bib-0003] Bitner, K. , Shahrestani, P. , Pardue, E. , & Mueller, L. D. (2020). Predicting death by the loss of intestinal function. PLoS One, 15(4), e0230970. 10.1371/journal.pone.0230970 32287318 PMC7156097

[ece37770-bib-0004] Borash, D. J. , Gibbs, A. G. , Joshi, A. , & Mueller, L. D. (1998). A genetic polymorphism maintained by natural selection in a temporally varying environment. The American Naturalist, 151, 148–156. 10.1086/286108 18811414

[ece37770-bib-0005] Borash, D. J. , Teotónio, H. , Rose, M. R. , & Mueller, L. D. (2000). Density‐dependent natural selection in *Drosophila*: Correlations between feeding rate, development time, and viability. Journal of Evolutionary Biology, 13, 181–187. 10.1046/j.1420-9101.2000.00167.x

[ece37770-bib-0006] Brown, E. B. , Slocumb, M. E. , Szuperak, M. , Kerbs, A. , Gibbs, A. G. , Kayser, M. S. , & Keene, A. C. (2019). Starvation resistance is associated with developmentally specified changes in sleep, feeding and metabolic rate. Journal of Experimental Biology, 222, 191049. 10.1242/jeb.191049 PMC6381993 30606795

[ece37770-bib-0007] Burnet, B. , Sewell, D. , & Bos, M. (1977). Genetic analysis of larval feeding rate behavior in *Drosophila melanogaster*. II. Genetical Research, 30, 149–161. 10.1017/s0016672300015196 4217753

[ece37770-bib-0008] Charnov, E. L. (1976). Optimal foraging: The marginal value theorem. Theoretical Population Biology, 9, 129–136. 10.1016/0040-5809(76)90040-X 1273796

[ece37770-bib-0009] Chippindale, A. K. , Chu, J. F. , & Rose, M. R. (1996). Complex trade‐offs and the evolution of starvation resistance in *Drosophila* . Evolution, 50, 753–766. 28568920 10.1111/j.1558-5646.1996.tb03885.x

[ece37770-bib-0010] Chippindale, A. K. , Hoang, D. T. , Service, P. M. , & Rose, M. R. (1994). The evolution of development in *Drosophila melanogaster* selected for postponed senescence. Evolution, 48, 1880–1899. 28565158 10.1111/j.1558-5646.1994.tb02221.x

[ece37770-bib-0011] Cody, M. (1966). A general theory of clutch size. Evolution, 20, 174–184. 10.1111/j.1558-5646.1966.tb03353.x 28563630

[ece37770-bib-0012] Dudley, S. A. , & Schmitt, J. (1996). Testing the adaptive plasticity hypothesis: Density‐dependent selection on manipulated stem length in *Impatiens* *capensis* . American Naturalist, 147, 445–465. 10.1086/285860

[ece37770-bib-0013] Fellowes, M. D. E. , Kraaijeveld, A. R. , & Godfray, H. C. J. (1999). Association between feeding rate and parasitoid resistance in *Drosophila melanogaster* . Evolution, 53, 1302–1305. 28565507 10.1111/j.1558-5646.1999.tb04544.x

[ece37770-bib-0014] Gadgil, M. , & Bossert, W. H. (1970). Life historical consequences of natural selection. American Naturalist, 104, 1–24. 10.1086/282637

[ece37770-bib-0015] Gibbs, A. G. , Chippindale, A. K. , & Rose, M. R. (1997). Physiological mechanisms of evolved desiccation resistance in *Drosophila melanogaster* . Journal of Experimental Biology, 200, 1821–1832. 10.1242/jeb.200.12.1821 9225453

[ece37770-bib-0016] Gilchrist, G. W. , & Huey, R. B. (1999). The direct response of *Drosophila melanogaster* to 571 selection on knockdown temperature. Heredity, 83, 15–29. 10.1038/sj.hdy.6885330 10447699

[ece37770-bib-0017] Guo, P. Z. , Mueller, L. D. , & Ayala, F. J. (1991). Evolution of behavior by density‐dependent natural selection. Proceedings of the National Academy of Sciences of the United States of America, 88, 10905–10906. 10.1073/pnas.88.23.10905 1961760 PMC53040

[ece37770-bib-0018] Joshi, A. , & Mueller, L. D. (1988). Evolution of higher feeding rate in *Drosophila* due to density‐dependent natural selection. Evolution, 42, 1090–1093. 28581181 10.1111/j.1558-5646.1988.tb02527.x

[ece37770-bib-0019] Joshi, A. , & Mueller, L. D. (1996). Density‐dependent natural selection in *Drosophila*: Trade‐offs between larval food acquisition and utilization. Evolutionary Ecology, 10, 463–474. 10.1007/BF01237879

[ece37770-bib-0020] Kaun, K. R. , Riedl, C. A. L. , Chakaborty‐Chatterjee, M. , Belay, A. T. , Douglas, S. J. , Gibbs, A. G. , & Sokolowski, M. B. (2007). Natural variation in food acquisition mediated via a *Drosophila* cGMP‐dependent protein kinase. Journal of Experimental Biology, 210, 3547–3558. 10.1242/jeb.006924 17921156

[ece37770-bib-0021] Kolss, M. , Vijendravarma, R. K. , Schwaller, G. , & Kawecki, T. J. (2009). Life history consequences of adaptation to larval nutritional stress in *Drosophila* . Evolution, 63, 2389–2401. 19473389 10.1111/j.1558-5646.2009.00718.x

[ece37770-bib-0022] Lewontin, R. C. (1974). The genetic basis of evolutionary change. Columbia University Press.

[ece37770-bib-0023] MacArthur, R. H. , & Pianka, E. R. (1966). On optimal use of a patchy environment. American Naturalist, 100, 603–609. 10.1086/282454

[ece37770-bib-0024] Mery, F. , & Kawecki, T. J. (2003). A fitness cost of learning ability in *Drosophila melanogaster* . Proceedings of the Royal Society of London, Series B: Biological Sciences, 270, 2465–2469. 14667336 10.1098/rspb.2003.2548 PMC1691529

[ece37770-bib-0025] Mueller, L. D. (1988a). Evolution of competitive ability in *Drosophila* due to density‐dependent natural selection. Proceedings of the National Academy of Sciences of the United States of America, 85, 4383–4386. 3132712 10.1073/pnas.85.12.4383 PMC280433

[ece37770-bib-0026] Mueller, L. D. (1988b). Density‐dependent population growth and natural selection in food limited environments: The *Drosophila* model. The American Naturalist, 132, 786–809. 10.1086/284890

[ece37770-bib-0027] Mueller, L. D. (1990). Density‐dependent natural selection does not increase efficiency. Evolutionary Ecology, 4, 290–297. 10.1007/BF02270928

[ece37770-bib-0028] Mueller, L. D. , & Barter, T. T. (2015). A model of the evolution of larval feeding rate in *Drosophila* driven by conflicting energy demands. Genetica, 143, 93–100. 10.1007/s10709-015-9818-5 25630626

[ece37770-bib-0029] Mueller, L. D. , Folk, D. G. , Nguyen, N. , Nguyen, P. , Lam, P. , Rose, M. R. , & Bradley, T. (2005). Evolution of larval foraging behaviour in *Drosophila* and its effects on growth and metabolic rate. Physiological Entomology, 30, 262–269.

[ece37770-bib-0030] Mueller, L. D. , & Joshi, A. (2000). Stability in model populations. Monographs in population biology. Princeton University Press.

[ece37770-bib-0031] Mueller, L. D. , Joshi, A. , Santos, M. , & Rose, M. R. (2013). Effective population size and evolutionary dynamics in outbred laboratory populations of *Drosophila* . Journal of Genetics, 92, 349–361. 10.1007/s12041-013-0296-1 24371158

[ece37770-bib-0032] Mueller, L. D. , Phillips, M. A. , Barter, T. T. , Greenspan, Z. , & Rose, M. R. (2018). Genome‐wide mapping of gene‐phenotype relationships in experimentally evolved populations. Molecular Biology and Evolution, 35, 2085–2095. 10.1093/molbev/msy113 29860403

[ece37770-bib-0033] Nagarajan, A. , Nagarajan, S. B. , Jayaram, M. , Thammanna, A. , Chari, S. , Bose, J. , Jois, S. V. , & Joshi, A. (2016). Adaptation to larval crowding in *Drosophila* *ananassae* and *Drosophila* *nasuta*: Increased larval competitive ability without increased larval feeding rate. Journal of Genetics, 95, 411–425. 27350686 10.1007/s12041-016-0655-9

[ece37770-bib-0034] Partridge, L. , & Fowler, K. (1992). Direct and correlated responses to selection on age at reproduction in *Drosophila melanogaster* . Evolution, 46, 76–91. 28564973 10.1111/j.1558-5646.1992.tb01986.x

[ece37770-bib-0035] Pinheiro, J. C. , & Bates, D. M. (2000). Mixed‐effects models in S and S‐PLUS. Springer.

[ece37770-bib-0036] R Core Team (2017). R: A language and environment for statistical computing. R Foundation for Statistical Computing. https://www.R‐project.org/

[ece37770-bib-0037] Rose, M. R. (1984). Laboratory evolution of postponed senescence in *Drosophila melanogaster* . Evolution, 38, 1004–1010. 28555803 10.1111/j.1558-5646.1984.tb00370.x

[ece37770-bib-0038] Rose, M. R. , Passananti, H. B. , & Matos, M. (2004). Methuselah flies: A case study in the evolution of aging. World Scientific Publishing.

[ece37770-bib-0039] Rose, M. R. , Vu, L. N. , Park, S. U. , & Graves, J. L. (1992). Selection for stress resistance increases longevity in *Drosophila melanogaster* . Experimental Gerontology, 27, 241–250. 1521597 10.1016/0531-5565(92)90048-5

[ece37770-bib-0040] Santos, M. , Borash, D. J. , Joshi, A. , Bounlutay, N. , & Mueller, L. D. (1997). Density‐dependent natural selection in *Drosophila*: Evolution of growth rate and body size. Evolution, 51, 420–432. 10.2307/2411114 28565346

[ece37770-bib-0041] Schlötterer, C. , Kofler, R. , Versace, E. , Tobler, R. , & Franssen, S. U. (2014). Combining experimental evolution with next‐generation sequencing: A powerful tool to study adaptation from standing genetic variation. Heredity, 114, 431–440. 10.1038/hdy.2014.86 25269380 PMC4815507

[ece37770-bib-0042] Service, P. M. , Hutchinson, E. W. , MacKinley, M. D. , & Rose, M. R. (1985). Resistance to environmental stress in *Drosophila melanogaster* selected for postponed senescence. Physiological Zoology, 58, 380–389. 10.1086/physzool.58.4.30156013

[ece37770-bib-0043] Sewell, D. , Burnet, B. , & Conolly, K. (1975). Genetic analysis of larval feeding behavior in *Drosophila melanogaster* . Genetics Research, 24, 163–173. 10.1017/s0016672300015196 4217753

[ece37770-bib-0044] Sinervo, B. (1990). The evolution of maternal investment in lizards: An experimental and comparative analysis of egg size and its effects on offspring performance. Evolution, 44, 279–294. 10.1111/j.1558-5646.1990.tb05198.x 28564384

[ece37770-bib-0045] Sokolowski, M. B. , Pereira, H. S. , & Hughes, K. (1997). Evolution of foraging behavior in *Drosophila* by density‐dependent selection. Proceedings of the National Academy of Sciences of the United States of America, 94, 7373–7377. 9207098 10.1073/pnas.94.14.7373 PMC23828

[ece37770-bib-0046] Stearns, S. C. (1992). The evolution of life histories. Oxford University Press.

